# The impact of frailty and malnutrition on hospitalisation and survival in people with kidney failure

**DOI:** 10.1007/s40620-025-02356-9

**Published:** 2025-07-23

**Authors:** Jessica Dawson, Brendan Smyth, Michele Ryan, Ann-Maree Randall, Katharine Clifford, Max Thomsett, Chenlei-Kelly Li, Mark A. Brown

**Affiliations:** 1 Dept Nutrition and Dietetics, St George Hospital, Sydney, NSW Australia; 2 Trials Centre, NHMRC Clinical, University of Sydney, Sydney, NSW Australia; 3 Dept of Renal Medicine, St George Hospital, Sydney, NSW Australia; 4 Western Renal Service, Sydney, Australia; 5 Dept of Nutrition and Dietetics, Nepean Hospital, Kingswood, Australia; 6 School of Clinical Medicine, George and Sutherland, University of New South Wales Medicine and Health, Kensington, Australia

**Keywords:** Frailty, Malnutrition, Conservative kidney management, Dialysis, Mortality, Hospitalization

## Abstract

**Background:**

Malnutrition and frailty are common, with rates increasing as kidney disease progresses and with ageing. Conservative kidney management has emerged as a kidney failure treatment that prioritises quality of life and symptom management, typically offered to the elderly. The aim of this study was to determine the incidence and impact of frailty and malnutrition on hospitalisations, quality of life and mortality in people with kidney failure.

**Methods:**

This two-year longitudinal study recruited people choosing conservative kidney management and, as a comparator group, people aged 75 years and over who were commenced on dialysis. Participants underwent assessment of frailty, nutritional status and quality of life every 6 months. Hospitalisation and death data were extracted from medical records.

**Results:**

A total of 85 participants were recruited (*n* = 60 conservative kidney management, *n* = 25 dialysis). At baseline, 56% were assessed as frail and 33% as malnourished. In the total cohort, frailty was associated with a higher rate of hospitalisations, and longer hospital stays. These associations appeared to be driven by the dialysis group, as no differences in hospitalisation rates or length of stay were observed in the conservative kidney management group based on frailty status. In the conservative kidney management group, frailty was not associated with mortality (HR 1.42, 95% CI 0.71–2.84; *p* = 0.3), however, being malnourished was associated with reduced 2-year survival (HR 2.10, 95% CI 1.13–3.90; *p* = 0.024).

**Conclusions:**

Frailty and malnutrition are common, resulting in adverse outcomes in elderly conservative kidney management and dialysis populations. Nutrition is a key intervention in both frailty and malnutrition, with clinical trials needed to evaluate safe and effective interventions.

**Graphical abstract:**

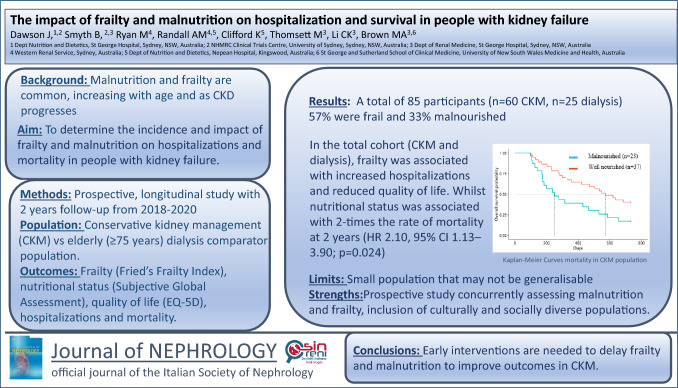

**Supplementary Information:**

The online version contains supplementary material available at 10.1007/s40620-025-02356-9.

## Introduction

Chronic kidney disease (CKD) is estimated to affect approximately 10% of the population [1] and is predicted to be the 5th leading cause of years of life lost by 2040 [2]. The prevalence of CKD increases with age, with an estimated 44% of Australians aged over 75 years being affected [1], and with almost half of the dialysis population aged over 65 years. [3] Frailty and malnutrition are common clinical conditions in the elderly and become increasingly prevalent as kidney disease progresses, and are independent predictors of mortality in CKD and dialysis populations [4–6].

Malnutrition and frailty are similar, yet distinct pathophysiologies that exacerbate each other and may have similar treatment strategies [7]. Frailty is defined as an age-related syndrome characterised by an increased state of vulnerability to stressors with cumulative deficits in many physiological systems [8]. Malnutrition (as related to undernutrition) is an imbalance between nutrient requirements and intake resulting in altered metabolism, impaired function and loss of body mass [9]. Both frailty and malnutrition are characterised by unintentional loss of body mass and impaired physical functioning. A defining difference between malnutrition and frailty is the cause of loss of body mass; whether it is due to inadequate nutritional intake or increased nutritional requirements (malnutrition) or due to hormonal, cytokine, or metabolic factors that may not respond to improvements in protein and energy intake (frailty). [10] Despite the high prevalence and overlap of malnutrition and frailty, there is a paucity of literature evaluating the co-existence and impact of these clinical conditions on outcomes for patients with kidney disease.

Recently there has been the emergence of conservative kidney management, a treatment pathway for (typically elderly) patients with kidney failure that focuses on reducing symptom burden and improving quality of life [11, 12], but does not include dialysis. Understanding the impacts of frailty and malnutrition in this population will help to target interventions to delay the progression of these conditions with a focus on improving the quality of life.

The aim of this 2-year longitudinal study was to determine the incidence and impact of frailty and malnutrition in elderly people with kidney failure at the initiation of conservative kidney management and dialysis. We have previously published findings regarding the association between upper gastrointestinal symptoms, dietary intake and frailty and malnutrition in this elderly cohort [13]. This study evaluates the impact of frailty and malnutrition on quality of life, hospitalisations, and mortality.

## Methods

Strengthening the Reporting of Observational Studies in Epidemiology (STROBE) guidelines were adhered to when preparing this manuscript. [14]

### Study design and setting

This was a prospective, observational study with a two-year follow-up. Written informed consent was obtained before enrolment into the study. This was a pragmatic study with data collected during clinic appointments by dietitians. Recruitment commenced across three renal units that serve socioeconomically and culturally diverse populations in Sydney, Australia from July 2018 to September 2020. Due to the COVID-19 pandemic physical distancing requirements limiting in-person consultation, recruitment was ceased early and data analysis was delayed due to Covid-19 deployment.

### Participants

Patients were eligible if they were commencing conservative kidney management (i.e., non-dialysis, medical management for kidney failure) or if they were aged 75 years or older and commencing dialysis. Given that frailty increases with age, an age eligibility criterion was included for people receiving dialysis in order to recruit a similarly aged cohort to that of the conservative kidney management cohort. There was no age requirement in conservative kidney management as people choosing conservative kidney management are typically elderly, with a mean age of 83 years. [15] People were excluded if they were withdrawing from dialysis, in the terminal phase of life (as per clinician assessment), currently hospitalised or were acutely unwell. People who met eligibility criteria were recruited at their initial conservative kidney management appointment or within 3 months of commencing dialysis. Participants continued to receive usual dietary counselling, including oral nutrition support when indicated.

### Outcomes

Frailty: was assessed using Fried’s Frailty Index [16]. Fried’s Frailty Index assesses frailty against five domains: shrinking (unintentional weight loss), weakness, slowness, exhaustion and low physical activity. As in previous studies [17], assessment criteria for level of physical activity was modified to reduce burden to participants and improve ease of collection (Supp File 1). Patients were classified as frail if they met 3 or more of the 5 criteria.

Nutritional status: was assessed using the 7-point Subjective Global Assessment [18]. The Subjective Global Assessment assesses the presence of undernutrition. Participants were classified as well-nourished if they scored ≥ 6 and were classified as malnourished if they scored ≤ 5.

Hospitalisations: hospital admissions and length of stay were extracted from medical records. Hospitalisation data do not include admissions for dialysis therapy or day stays.

Quality of life: EuroQoL five dimensions (EQ-5D) is routinely collected on patients every 6 months when attending conservative kidney management clinics. EQ-5D domains are rated on a scale from 1 (no deficit) to 5 (extreme deficit) and a scale from 0–100 rating health status, with higher scores indicating better health. [19]

Survival: date and cause of death were extracted from medical records.

Other outcomes measured included dietary intake (assessed using 24-h pass methodology [20], gastrointestinal symptom burden (determined using the iPOS-Renal survey completed at the time of assessment) and biochemical parameters.

### Statistics

Variables are presented as means and standard deviations or median (IQR) for non-normally distributed data. Variables with a normal distribution were compared between groups using Student’s t-test, or Mann–Whitney test for non-normally distributed variables. The chi square test was used to compare categorical variables. ETA-squared correlational analyses were used to determine correlation between nominal data and categorical data. Survival was assessed using the Kaplan–Meier method. Negative binomial regression analysis to investigate predictors of hospitalisation was conducted with a priori covariates including age, gender, presence of diabetes, presence of congestive cardiac failure, nutritional status, and frailty. Based on assumed rates of frailty of 42% in CKD and 67% in dialysis populations, a sample size of 60 participants in each cohort ( conservative kidney management and dialysis) was determined to be needed to detect a between-group difference in the rate of frailty with 80% power with an alpha 0.05. Statistical analysis was performed using R and SPSS (v28.0.0.0). A significance level of < 0.05 was used.

## Results

A total of 85 people who were receiving conservative kidney management (*n* = 60) or were aged > 75 years and were commencing dialysis (*n* = 25) were recruited. The dialysis cohort was predominantly commencing Peritoneal Dialysis (*n* = 23, 92%), in keeping with the participating renal units’ dialysis policy. Recruitment into the dialysis cohort ceased early due to slower recruitment owing to the impact from COVID-19. The mean age of the total cohort was 80 years, 62% were male (*n* = 53), 35% of participants were born in Australia (*n* = 30) and 66% (*n* = 56) reported their primary language as English (Table 1 Baseline characteristics). There was no difference in baseline characteristics between conservative kidney management and dialysis cohorts (Supp File 2). Overall, 48 participants (56%) were assessed as frail and 28 participants (33%) were malnourished. Rates of frailty and malnutrition were higher in the conservative kidney management cohort than the dialysis cohort, however this did not reach statistical significance (62% vs 44% frailty, *p* = 0.18 and 38% vs 20% malnutrition, *p* = 0.10, respectively).

**Table 1:** Participant Baseline

**Characteristics**	**Total cohort** (*n* = 85)
Sex, Male *n* (%)	53 (62%)
Age mean (SD)	80.7 (10.6)
Primary language, English n (%)	56 (66%)
Country of Birth, Australia n (%)	30 (35%)
**Co-morbidities**, *n* (%)	
Total number (mean, SD)	4.7 (2.5)
Diabetes	46 (54%)
Cardiovascular disease	52 (61%)
Congestive Cardiac Failure	15 (18%)
**Biochemistry**	
Urea	25.2 (8.4)
Creatinine	440 (174)
eGFR	11 (5)
Bicarbonate	22.8 (3.8)
Albumin	33.6 (5.7)
Potassium	4.6 (0.7)
Phosphate	1.62 (0.38)
Haemoglobin	107.2 (16.1)
**Frailty status**	
Frail	48 (57%)
Weight loss	31 (36%)
Weakness	66 (78%)
Slow walking	19 (22%)
Exhaustion	36 (42%)
Low physical activity	71 (84%)
**Nutritional status**	
Malnourished	28 (33%)
**Anthropometry**	
Weight, median (IQR)	72.6 kg (61.4–80.6)
Body Mass Index, median (IQR)	26 kg/m2 (23.7–29.8)
**Gastrointestinal symptoms**	
Poor appetite, *n* (%)	41 (48%)
Dry or sore mouth, *n* (%)	56 (66%)
Taste changes, *n* (%)	30 (35%)
Nausea, *n* (%)	14 (16%)
Vomiting, n (%)	6 (7%)
Constipation, *n* (%)	29 (34%)
Diarrhoea, *n* (%)	9 (11%)
Mean number of symptoms	2.8 (2.0)
Symptom severity	1.5 (0.8)
**Dietary intake**	
Energy (kcal/day), median (IQR)	1370 kcal (1111–1557)
Energy (kcal/kg/day), median (IQR)	19 (16–23)
Protein (g/day), median (IQR)	67 g (5–80)
Protein (g/kg/day), median (IQR)	1 g/kg/d (0.7–1.2)

g: grams, kg: kilograms

Participants had a median of 2 (IQR 1–3) gastrointestinal symptoms and median gastrointestinal symptom severity score of 1.3, indicating symptoms to be slight to moderate in severity. The most frequently reported gastrointestinal symptoms were sore/dry mouth (*n* = 56, 66%), anorexia (*n* = 41, 48%) and taste changes (*n* = 30, 35%). Compared to not frail participants, those assessed as frail reported significantly higher rates of dry/sore mouth (46% vs 76%, *p* = 0.006) and anorexia (30% vs 58%, *p* = 0.01). Being malnourished (OR 5.6, 95% CI 1.4, 2.17, *p* = 0.013) and having more severe gastrointestinal symptoms (OR 2.8, 95% CI 1.1, 7.0, *p* = 0.025) predicted frailty. Similarly, compared to participants who were well nourished, those who were assessed as being malnourished reported higher rates of anorexia (32% vs 82%, *p* < 0.001), dry/sore mouth (56% vs 86%, *p* = 0.007) and taste changes (28% vs 50%, *p* = 0.047). Malnourished participants consumed significantly less protein (0.77 g/kg/day vs 1.05 g/kg/day, *p* < 0.001) and fewer calories (1235 kcal/day vs 1400 kcal/day, *p* = 0.01). [13]

### Hospital admissions

In the total cohort, fifty-four participants (63.5%) had one or more hospital admissions over the 2-year follow-up. There was no difference in the proportion of conservative kidney management and dialysis participants requiring at least one hospital admission (65% and 60%, respectively; *p* = 0.663), nor the number of admissions (median 1 (IQR 0–2) in conservative kidney management vs median 1 (IQR 0–2) in dialysis, *p* = 0.76) or length of stay (median 6 days (IQR 0–20.5) in conservative kidney management vs median 8.6 days (IQR 0–31.4) in dialysis; *p* = 0.73) (Table 2). Similarly, in the total cohort there was no difference in the proportion of participants requiring at least one hospital admission, number of hospital admissions nor length of stay when analysing by nutritional status (malnourished vs well nourished) (Table 2). However, participants who were assessed as being frail had significantly more hospital admissions than those who were not frail (median 1 (IQR 0–2) vs median 0 (IQR 0–2); *p* = 0.027, respectively) and had significantly longer length of stay compared to those who were not frail (median 12 days (IQR 0–24) vs median 0 days (IQR 0–3), *p* = 0.004, respectively) (Table 2). Frailty predicted hospitalisation independently of age, sex, diabetes, and history of congestive cardiac failure, (incident rate ratio (IRR) 1.8 (95% CI 1.07, 3.2) *p* = 0.029), although its impact was attenuated when malnutrition was included in the model (Supp File 3).

**Table 2:** Rates of hospitalisation by treatment pathway, frailty and nutritional status

	% requiring ≥ 1 admission		Number of hospital admissions		Length of stay (days)	
Total cohort (*n* = 85)						
CKM (*n* = 60)	39 (65%)	*P* = 0.63	1 (0, 2)	*P* = 0.76	6 (0, 20.5)	*P* = 0.73
Dialysis (*n* = 25)	15 (60%)		1 (0, 2)		8.6 (0, 31.4)	
Frail (*n* = 55)	40 (73%)	***P* = 0.017**	1 (0, 2)	***P* = 0.027**	12 (0, 24)	***P* = 0.004**
Not frail (*n* = 30)	14 (47%)		0 (0, 2)		0 (0, 3)	
Malnourished (*n* = 28)	20 (71%)	*P* = 0.29	2 (0, 2.5)	*P* = 0.13	9.8 (0, 26)	*P* = 0.22
Well nourished (*n* = 57)	34 (60%)		1 (0, 2)		2 (0, 18)	
CKM (*n* = 60)						
Frail (*n* = 42)	29 (69%)	*P* = 0.32	1 (0, 2)	P = 0.16	9 (0, 21)	*P* = 0.16
Not frail (*n* = 18)	10 (56%)		1 (0, 2)		1.5 (0, 15)	
Malnourished (*n* = 23)	15 (65%)	*P* = 0.98	1 (0, 2)	P = 0.85	7 (0, 19)	*P* = 0.85
Well nourished (*n* = 37)	24 (65%)		1 (0, 2)		3 (0, 21)	
Dialysis (*n* = 25)						
Frail (*n* = 13)	11 (85%)	***P* = 0.009**	2 (2, 4)	***P* = 0.003**	18 (13, 43)	***P* = 0.006**
Not frail (*n* = 12)	4 (33%)		0 (0, 1)		0 (0, 1.5)	
Malnourished (*n* = 5)	5 (100%)	***P* = 0.041**	2 (2, 4)	***P* = 0.028**	31 (17.5, 54.4)	***P* = 0.025**
Well nourished (*n* = 20)	10 (50%)		0.5 (0, 2)		5 (0, 17.5)	

Bolded *p*-values denote significance as *p* < 0.05

Data presented as n (%) and median (IQR).   Abbreviations; CKM; Conservative Kidney Management

For patients on a conservative kidney management pathway there was no significant difference in hospitalisation based on frailty (55.5% not frail, 69% frail, *p* = 0.315) or nutritional status (65% well nourished, 65% malnourished, *p* = 0.978). This included no significant difference in the number of admissions, nor the length of stay by either frailty or nutritional status (Table 2). However, for participants receiving dialysis, there was a significantly higher proportion requiring at least one hospital admission based on frailty (33% for those not frail, 85% for those frail, *p* = 0.009) and nutritional status (50% for those well-nourished, 100% for those malnourished, *p* = 0.041). There were also significantly higher rates of hospitalisation and length of stay based on frailty and nutritional status in the dialysis cohort (Table 2).

### Quality of life

Quality of life data were only available for a subset of conservative kidney management participants (*n* = 51, 85% of the total conservative kidney management cohort). Participants who were assessed as being frail rated their overall health status as lower than those who were not frail (median 50 (IQR 50–70) vs median 82.5 (IQR 55–90), respectively; *p* = 0.012), but there was no statistically significant difference in self-reported health status of those who were assessed as being malnourished compared to well-nourished participants (median 50 IQR 50–60; median 70 IQR 50–75 *p* = 0.082). Compared to conservative kidney management participants who were not frail, frail conservative kidney management participants reported having significantly greater deficits (rated from slight to extreme) in mobility (*p* < 0.001), ability to self-care (*p* = 0.002), participation in usual activities (*p* = 0.007), pain (*p* = 0.012) and anxiety/depression (*p* = 0.028) (Supp Files 4 & 5). In the conservative kidney management subset, frailty was strongly correlated with self-reported health status (r = 0.405, *p* = 0.005), whilst malnutrition was weakly correlated with self-reported health status (r = 0.235, *p* = 0.116).

### Mortality

There was an overall median survival of 656 days, with 47% survival at 2 years. Of the total cohort, people who were assessed as frail at baseline (*n* = 55, 65%) had a median survival of 549 days, whilst those who were not frail had a median survival > 2 years. At 2 years, 41% of people assessed as frail and 57% of those who were not frail were alive (*p* = 0.12). The median survival of people assessed as being malnourished (*n* = 28, 33%) was 372 days compared to > 2 year median survival in those well-nourished. At 2 years, 54% of those who were well nourished were alive, compared to 32% of people who were malnourished (*p* = 0.016).

When analysed by treatment for kidney failure ( conservative kidney management or dialysis), 84% of people on dialysis were alive at 2 years, compared to 30% of people receiving conservative kidney management (*p* < 0.001). People choosing conservative kidney management had a shorter survival time of 468 days than those receiving dialysis who had a median survival > 2 years (*p* < 0.001) (Supp File 6).

### Impact of frailty and malnutrition on survival in the conservative kidney management cohort only

**Fig. 1:**
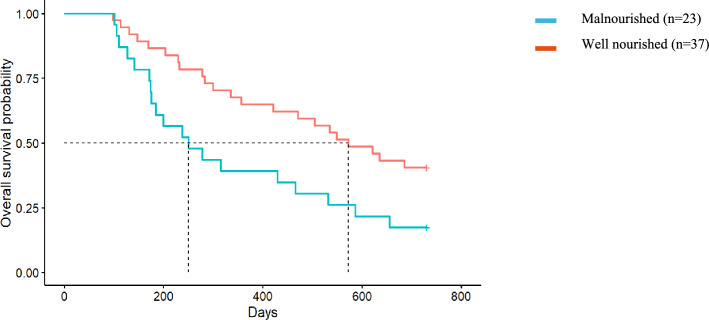
Malnutrition and survival in the Conservative Kidney Management population. Median survival for those who were malnourished was 250 days compared to those who were well nourished with a median of 18.8 months (Hazard Ratio = 2.10, 95% CI 1.13, 3.90, *p* = 0.02)

**Fig. 2:**
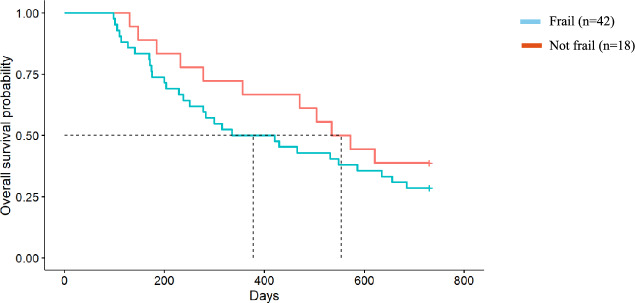
Frailty and survival in the Conservative Kidney Management cohort. Median survival for those who were frail was 12.4 months compared to those who were not frail with a median survival of 18.2 months (Hazard Ratio = 1.42, 95% CI 0.71, 2.84, *p* = 0.3)

The median survival of dialysis participants exceeded 2 years, therefore the effect of malnutrition and frailty on survival of people choosing conservative kidney management only (*n* = 60) was analysed. People receiving conservative kidney management who were malnourished at baseline survived on average 250 days (95% CI 128, 372) (equivalent to 8.2 months) compared with an average 572 days (95% CI 415, 728) (equivalent to 18.8 months) in well-nourished patients (*p* = 0.021) and had twice the rate of death as those who were well nourished (HR 2.10, 95% CI 1.13, 3.90; *p* = 0.024) (Fig. 1). Participants who were frail at baseline had a 40% increased rate of death compared to those who were not frail, although this did not reach statistical significance (HR 1.42, 95% CI 0.71, 2.84; *p* = 0.3) (Fig. 2). The median survival for people choosing conservative kidney management who were frail at baseline was an average of 335 days (95% CI 141, 529) (equivalent to 11 months) compared to 535 days (95% CI 393, 676) (equivalent to 17.5 months) for those who were not frail (*p* = 0.26).

## Discussion

The key findings in this study are that in elderly patients with kidney failure, frailty is associated with a greater likelihood of hospitalisation and longer length of stay. In the conservative kidney management population, participants who were frail had poorer quality of life, whilst malnutrition was associated with poorer 2-year survival in conservative kidney management. However, there was no difference in hospitalisations or length of stay based on frailty or malnutrition in the conservative kidney management population. Conversely, in the elderly dialysis population, frailty and malnutrition were associated with higher rates of hospitalisation and longer length of stay.

To our knowledge, this is the first study to evaluate the impact of both frailty and malnutrition on an elderly kidney failure population. Frailty and malnutrition are interrelated and overlapping constructs, both of which are well-established risk factors for adverse patient events, including increased rates of hospitalisation [21, 22]. However in this study, there was no association of frailty or malnutrition with hospitalisation in the conservative kidney management population. One potential reason is that renal units that participated in this study have established nurse-led kidney supportive care clinics that provide regular follow up to patients choosing conservative kidney management that includes symptom management, dietary and social care to support patients to remain at home. Multidisciplinary care in CKD improved patient outcomes and resulted in an estimated 48% reduction in the risk of hospitalisation and 33% reduction in all-cause mortality [23]. Whilst it cannot be confirmed from this study, regular access to kidney supportive care multidisciplinary teams may pre-empt and manage adverse events that often result in hospitalisations, including those who are frail or malnourished, who are most at risk.

The rate of frailty (57%) in this study is consistent with previous literature showing rates of frailty of 47–73% in kidney failure populations [4, 24], although most of these other studies have been conducted in younger cohorts (< 65 years). [4] King et al. reported a mean survival of 3.48 years with frail participants having a 13-month shorter survival when compared to non-frail people with advanced CKD [24]. The survival of this elderly cohort [24] was longer than in our patients, likely as theirs was a younger elderly population (mean age 76.3 years vs mean age 80.7 years) who had higher estimated glomerular filtration rate (eGFR) at baseline (16 ml/min/1.73m^2^ vs 11 ml/min/1.73m^2^) and overall lower rate of frailty (47% vs 57%).

An interesting finding in our study was the lack of a significant association of malnutrition and quality of life.

in conservative kidney management participants. These results differ from other studies that have reported malnutrition being associated with reduced quality of life in dialysis [25, 26] and transplant populations [26]. Viramontes-Horner et al [25] reported that in a dialysis-dependent population (*n* = 150), malnutrition was associated with a reduction in all domains of quality of life (mobility, self-care, usual activities, pain, anxiety/depression). This younger dialysis-dependent population reported similar health ratings to that of our elderly cohort among those who were assessed as well-nourished and those assessed as malnourished. Similarly, our study did not find a significant difference in hospitalisations based on nutritional status, although the increased length of stay is likely to be clinically important (median 9.8 days vs median 2 days). In a large CKD cohort (*n* = 682), being malnourished significantly increased length of hospital stay (median 13 vs 9 days in well-nourished participants). [27]

Malnutrition is a well-established predictor of mortality, with this being reflected in our elderly conservative kidney management population showing a 2.2-fold increased rate of 2-year mortality, equivalent to approximately 10 months shorter survival. Factors affecting malnutrition in this elderly kidney failure population have not been identified, however high gastrointestinal symptom burden, reduced oral intake and inflammation have been attributed to the development of malnutrition in dialysis populations [5]. These factors have been reflected in our elderly kidney failure cohort that showed higher gastrointestinal symptom burden and reduced oral intake to be significantly associated with frailty and malnutrition [13]. Our findings suggest that early nutrition assessment and counselling, prior to the commencement of dialysis or conservative kidney management, that includes a thorough symptom assessment, needs to be incorporated into routine care to identify people at risk of frailty and malnutrition. This early identification will enable timely interventions, including dietary counselling and/or oral nutrition support to increase caloric and protein intake when indicated, to delay the progression of these conditions.

There remains equipoise with respect to protein requirements and recommendations in elderly kidney failure populations. Dietary guidelines for CKD recommend limiting protein intake to ≤ 0.8 g protein/kg/day to reduce the build-up of uraemic toxins and slow CKD progression [28]. However, protein studies in CKD to date have failed to include elderly participants, limiting certainty and application of these recommendations to this growing kidney failure population. Adding to the uncertainty, guidelines for frailty, sarcopenia and malnutrition recommend 1–1.5 g protein/kg/day [29]. A large observational study in elderly people with CKD stages 1–3 (*n* = 4789) reported a linear trend for lower mortality with increasing total protein intake [30]. Compared to 0.8 g protein/kg/day, every 0.2 g/kg protein increase was associated with improved survival, and there was no difference in survival when comparing plant protein and animal protein intake [30]. Whether lower protein diets are safe or beneficial in elderly CKD populations remains unclear and needs further investigation. Additionally, patient preferences regarding dietary intake needs to be considered, particularly in conservative kidney management populations where symptom management and quality of life are often prioritised above interventions to prolong survival.

An important consideration alongside protein intake is that of adequate energy intake to maintain neutral nitrogen balance. In the observational study of early CKD [30], participants were consuming a total energy intake of approximately 28 kcal/kg/day, higher than the intake in the current study (approximately 20 kcal/kg/day). Resting energy expenditure in elderly people is estimated at approximately 20 kcal/kg/day, with requirements of 27–30 kcal/kg/day depending on level of physical activity [31]. The energy intake was low across our entire cohort, irrespective of frailty or nutritional status, and may be due to underestimation in dietary recalls. Dietary interventions need to consider both energy and protein when assessing nutritional adequacy, particularly in the frail elderly where there is reduced protein utilisation. Clinical trials evaluating the impact of dietary interventions, including optimal protein and energy prescription, on malnutrition and frailty in elderly people with kidney failure are needed.

The strengths of this study include its prospective design conducted across socioeconomic and culturally diverse renal units. This study is one of the first to assess both the association of malnutrition and frailty with hospitalisation, quality of life and mortality in an elderly CKD population. Additionally, this is one of the first studies to evaluate the impact of frailty and malnutrition in people on a conservative kidney management pathway, providing novel evidence highlighting the co-existing and compounding nature of these conditions that reduce quality of life, increase hospitalisations and reduce mortality. A study limitation is that most dialysis participants were on peritoneal dialysis, which is not representative of the broader dialysis population who predominantly receive haemodialysis, limiting generalisability. Further, it is recognised that dietary recall methodologies often underestimate intake and whilst the validated 24-h pass methodology was used, as several dietitians were involved in conducting dietary assessments there may have been inter-rater variability. Finally, both frailty and malnutrition were assessed using validated, evidence-based tools, however both required in-person physical assessments that was disrupted by the COVID-19 pandemic. As such, enrolment was ceased early, resulting in a small cohort that limits generalisability and statistical power that may have resulted in type II errors, particularly in the subgroup analyses.

Frailty and malnutrition are common in elderly people with kidney failure. Whilst frailty was associated with reduced quality of life and increased rate of hospitalisation and length of stay, malnutrition was associated with reduced survival, translating to 10 months shorter survival. Frailty and malnutrition were not associated with hospitalisations in the conservative kidney management population, and the impact of multidisciplinary care, such as the kidney supportive care model, on hospitalisations and adverse patient outcomes warrants further investigation. There remains uncertainty in how to optimally manage frailty and malnutrition. The observed overlap between frailty and malnutrition underscores the importance of integrated assessment and management strategies targeting both conditions. Nutrition is a key intervention for both conditions; however, it is unclear how to integrate the current protein guidelines for CKD and frailty/malnutrition in the elderly kidney failure population. Future trials are needed to evaluate effective and safe nutrition interventions for elderly people living with kidney failure, particularly those who have chosen conservative kidney management.

## Supplementary Information

Below is the link to the electronic supplementary material.

Supplementary file1 (DOCX 56 KB)

## Data Availability

The datasets generated and/or analysed during the current study are available from the corresponding author on reasonable request.
