# Correction: The impact of frailty and malnutrition on hospitalisation and survival in people with kidney failure

**DOI:** 10.1007/s40620-025-02407-1

**Published:** 2025-08-12

**Authors:** Jessica Dawson, Brendan Smyth, Michele Ryan, Ann-Maree Randall, Katharine Clifford, Max Thomsett, Chenlei-Kelly Li, Mark A. Brown

**Affiliations:** 1 Dept Nutrition and Dietetics, St George Hospital, Sydney, NSW Australia; 2 Trials Centre, NHMRC Clinical, University of Sydney, Sydney, NSW Australia; 3 Dept of Renal Medicine, St George Hospital, Sydney, NSW Australia; 4 Western Renal Service, Sydney, Australia; 5 Dept of Nutrition and Dietetics, Nepean Hospital, Kingswood, Australia; 6 School of Clinical Medicine, George and Sutherland, University of New South Wales Medicine and Health, Kensington, Australia

**Correction: Journal of Nephrology (2025)** 10.1007/s40620-025-02356-9

In this article, the first name and family name of all authors have been incorrectly swapped in the original published article. The complete correct name are given below.

Jessica Dawson, Brendan Smyth, Michele Ryan, Ann-Maree Randall, Katharine Clifford, Max Thomsett, Chenlei-Kelly Li, Mark A Brown.

The original article has been corrected.

