# Supplementary material for: The impact of frailty and malnutrition on hospitalisation and survival in people with kidney failure
Source: J Nephrol. 2025 Jul 23;38(8):2251–9. doi: 10.1007/s40620-025-02356-9 (PMC12630193; doi:10.1007/s40620-025-02356-9)
Supplement: Supplementary file 1 — Supplementary file1 (DOCX 56 KB) [file 40620_2025_2356_MOESM1_ESM.docx]

**Supplementary File 1: FFI assessment criteria**

| **Domain** | **Assessment Criteria** |
| --- | --- |
| Shrinking | Unintentional weight loss of >4.5kg in the past 12 months |
| Weakness | Hand Grip Strength being in the lowest 20^th^ percentile of a community-dwelling population of adults aged 65 years and older. Grip strength was assessed using a handheld dynamometer (Jamar Plus, Sammsons Preston Rolyan, Bolingbrook, IL, USA). The participant was asked to use their dominant hand and the best of three attempts was recorded. |
| Slowness | Four meter walking test. Individuals with a walking speed of less than the 20^th^ percentile of a community-dwelling elderly population, adjusted for height and body mass index. |
| Exhaustion | Two items from the Center for Epidemiological Studies Depression Scale (CES-D) were used.   1. How often did you feel everything you did was an effort? 2. How often did you feel you could not get going.   Participants were asked how often in the past week they felt this way. Those who answered much of the time / almost all the time to one of these questions was determined to be frail. |
| Low physical activity levels | Assessed using self-reported physical activities being light, moderate or strenuous. Those who reported hardly / never to moderate or strenuous physical activity were assessed to meet frailty criteria. |

**Supplementary File 2: Baseline characteristics by treatment modality**

| **Variables** | **CKM (n=60)** | **Dialysis (n=25)** | **p-value** |
| --- | --- | --- | --- |
| Age mean (SD) | 80.9 (12.5) | 80.2 (3.3) | 0.09 |
| Sex, male n (%) | 36 (60%) | 17 (68%) | 0.49 |
| Primary language, English n (%) | 39 (65%) | 17 (68%) | 0.79 |
| Country of Birth, Australia n (%) | 25 (42%) | 5 (20%) | 0.06 |
| Comorbidities, n (%) | 4.5 (2.7) | 5.1 (2) | 0.32 |
| Malnourished, n (%) | 23 (38%) | 5 (20%) | 0.10 |
| Frail, n (%) | 37 (62%) | 11 (44%) | 0.18 |

**Supplementary File 3: Predictors of Hospitalization**

| **Predictors of Hospitalization** | | | | | | | | |
| --- | --- | --- | --- | --- | --- | --- | --- | --- |
| **Variable** | **Model 1** |  | **Model 2** |  | **Model 3** |  | **Model 4** |  |
|  | IRR (95% CI) | p-value | IRR (95% CI) | p-value | IRR (95% CI) | p-value | IRR (95% CI) | p-value |
| Frailty | 1.8 (1.07, 3.15) | **0.027** | 1.94 (1.11, 3.37) | **0.019** | 1.85 (1.07, 3.21) | **0.029** | 1.66 (0.94, 2.94) | 0.08 |
| Age |  |  | 0.98 (0.94, 1.02) | 0.33 | 0.99 (0.95, 1.03) | 0.49 | 0.99 (0.95, 1.03) | 0.63 |
| Gender, male |  |  | 1.02 (0.61, 1.7) | 0.94 | 1.08 (0.65, 1.8) | 0.77 | 1.08 (0.65, 1.8) | 0.76 |
| Diabetes |  |  |  |  | 1.3 (0.79, 2.16) | 0.28 | 1.3 (0.79, 2.11) | 0.31 |
| CCF |  |  |  |  | 0.7 (0.35, 1.4) | 0.32 | 0.72 (0.36, 1.42) | 0.34 |
| Malnutrition |  |  |  |  |  |  | 1.4 (0.8, 2.3) | 0.23 |
| IRR: Incident Rate Ratio; CCF: congestive cardiac failure  Model 1: unadjusted; Model 2: adjusted for age and sex; Model 3: model 2+ adjusted for diabetes, CCF; Model 4: Model 3 + adjusted for malnutrition | | | | | | | | |

**Supplementary File 4: Quality of life domains reported in CKM cohort**

| **Quality of life domains reported in CKM cohort** | | | | |
| --- | --- | --- | --- | --- |
|  | **Frailty** | | **Nutritional status** | |
|  | **Not frail** | **Frail** | **Well-nourished (n=31)** | **Malnourished**  **(n=20)** |
| EQ-5D mobility | 1.0 (1.0, 3.0) | 3.0 (2.0, 4.0) | 2.0 (1.5, 3) | 3.0 (2, 4) |
| EQ-5D self-care | 1.0 (1.0, 1.0) | 2.0 (1.0, 3.0) | 1.0 (1.0, 3.0) | 1.0 (1.0, 3.0) |
| EQ-5D usual activities | 1.0 (1.0, 2.0) | 2.5 (2.0, 4.0) | 2.0 (1.0, 3.0) | 2.50 (1.5, 5.0) |
| EQ-5D pain | 1.0 (1.0, 2.0) | 2.5 (2.0, 3.0) | 2.0 (1.0, 3.0) | 3.0 (1.5, 3.0) |
| EQ-5D anxiety | 1.0 (1.0, 2.0) | 2.0 (1.0, 3.0) | 2.0 (1.0, 2.0) | 2.0 (1.0, 3.0) |
| EQ-5D Scale | 82.50 (55, 90) | 50.0 (50, 60) | 70.0 (50, 75) | 50.0 (50, 60) |
| Data expressed as median (IQR)  EQ-5D domains are scored from 0 (no deficit) to 5 (extreme deficits) | | | | |

**Supplementary File 5: Proportion of participants reporting deficits in quality of life domain**

Quality of life domains, participants reporting slight to extreme deficits. Data presented as percentages (%), represents the proportion of population reporting slight to extreme deficits in domains of quality of life.

**Supplementary File 6: Kaplan-Meier curves showing 2-year survival by kidney failure treatment pathway**


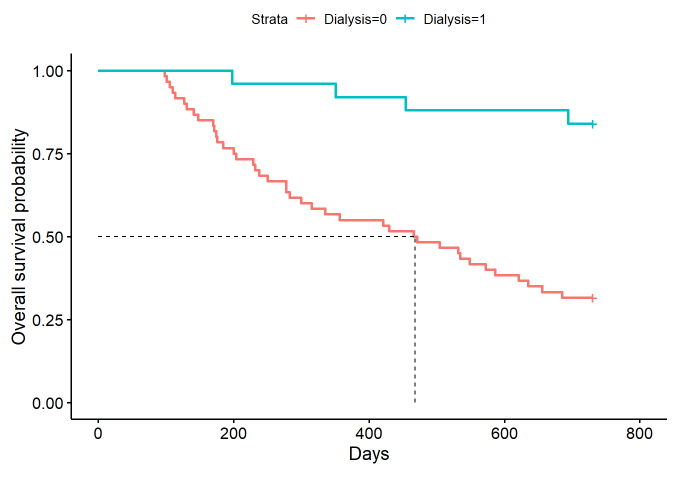


Dialysis (n=25)

CKM (n=60)

Kaplan-Meier curve showing rates of survival by kidney failure treatment with CKM having a median 468 days (95% CI 300, 635) and dialysis having a median survival greater than 2 years (p<0.001).
